# Screening of West Siberian patients with primary congenital glaucoma for CYP1B1 gene mutations

**DOI:** 10.18699/VJ20.684

**Published:** 2020-12

**Authors:** D.E. Ivanoshchuk, S.V. Mikhailova, O.G. Fenkova, E.V. Shakhtshneider, A.Z. Fursova, I.Y. Bychkov, M.I. Voevoda

**Affiliations:** Institute of Cytology and Genetics of Siberian Branch of the Russian Academy of Sciences, Novosibirsk, Russia; Institute of Cytology and Genetics of Siberian Branch of the Russian Academy of Sciences, Novosibirsk, Russia; Novosibirsk State Regional Hospital, Novosibirsk, Russia; Institute of Cytology and Genetics of Siberian Branch of the Russian Academy of Sciences, Novosibirsk, Russia; Institute of Cytology and Genetics of Siberian Branch of the Russian Academy of Sciences, Novosibirsk, Russia; S.N. Fyodorov FSBI IRTC Eye Microsurgery, Novosibirsk branch, Novosibirsk, Russia; Institute of Cytology and Genetics of Siberian Branch of the Russian Academy of Sciences, Novosibirsk, Russia

**Keywords:** human, congenital glaucoma, CYP1B1, genetic analysis, cytochrome P450 1B1, человек, врожденная глаукома, CYP1B1, генетический анализ, цитохром P450 1B1

## Abstract

Primary congenital glaucoma (PСG) is a visual organ pathology that leads to progressive blindness and poor vision in children. Its main cause is an anomaly of the anterior chamber angle. Most cases of PСG are sporadic, but familial cases with an autosomal recessive (predominantly) and autosomal dominant (rare) type of inheritance have been described. Congenital glaucoma is a rare condition (1 case per 10,000–20,000 newborns), but its prevalence is substantially higher (up to 1 case per 250 newborns) in countries where consanguineous marriages are common. Mutations in the CYP1B1 gene, which encodes cytochrome P450 1B1, are the most common cause of autosomal recessive primary congenital glaucoma. This enzyme is known to be involved in retinoic acid metabolism and is necessary for normal eye development. The aim of this work was to assess the polymorphism of the CYP1B1 gene among West Siberian patients with primary congenital glaucoma. Direct automatic Sanger sequencing of exons and adjacent splicing sites of the CYP1B1 gene was carried out in 28 people with the PCG phenotype from a West Siberian region. As a result, in the sample of the white population we examined, pathogenic variants previously described in other ethnic groups were revealed: E387K (rs55989760), R444* (rs377049098), R444Q (rs72549376), and P437L (rs56175199), as well as novel single-nucleotide deletion p.F114Lfs*38 in the CYP1B1 gene. The latter can cause a frame shift, changed amino acid composition, and a formation of truncated in the protein. None of the detected mutations were found in the control sample of ophthalmologically examined individuals without PCG (100 people). Variants R444* (rs377049098) and R444Q (rs72549376) were not found in the general population sample either (576 randomly selected West Siberia residents). All the detected mutations caused the development of the autosomal recessive form of primary congenital glaucoma. The most severe clinical phenotype was observed in carriers of mutations in codon 444 of the gene. Consequently, in children with signs of increased intraocular pressure, molecular genetic analysis of the CYP1B1 gene is advisable for early diagnosis and timely initiation of PCG therapy.

## Abstract (ru)

Первичная врожденная глаукома – это патология органа зрения, которая приводит к прогрессирующей слепоте и слабовидению у детей. Основной ее причиной являются аномалии/нарушения развития угла передней камеры глаза. Большинство случаев возникновения первичной врожденной глаукомы спорадические, но описаны и семейные варианты с аутосомно-рецессивным (преимущественно) и аутосомнодоминантным (редко) типом наследования. Врожденная глаукома – редкое заболевание (один случай на 10 000–20 000 новорожденных), но частота ее возникновения существенно возрастает (до одного случая на 250 новорожденных) в странах, где распространены близкородственные браки. Мутации в гене CYP1B1, который кодирует цитохром P450 1B1, становятся наиболее частой причиной аутосомно-рецессивных форм первичной врожденной глаукомы. Известно, что этот фермент участвует в метаболизме ретиноевой кислоты и необходим для нормального развития глаза. Целью нашей работы была оценка полиморфизма гена CYP1B1 у пациентов Западной Сибири с первичной врожденной глаукомой. Было проведено прямое автоматическое секвенирование по Сэнгеру экзонов и прилегающих сайтов сплайсинга гена CYP1B1 у 28 человек из Западно-Сибирского региона с фенотипом первичной врожденной глаукомы. В результате в обследованной нами выборке европеоидного населения выявлены ранее описанные в других этнических группах патогенные варианты этого гена: E387K (rs55989760), R444 * (rs377049098), R444Q (rs72549376) и P437L (rs56175199), а также новая однонуклеотидная делеция, приводящая к сдвигу рамки считывания p.F114Lfs*38 в гене CYP1B1. Последняя может вызывать образование белка с измененным аминокислотным составом и укороченного белка. Ни одна из выявленных мутаций не была выявлена в контрольной выборке офтальмологически обследованных лиц без первичной врожденной глаукомы (100 человек). Варианты R444* (rs377049098) и R444Q (rs72549376) не были найдены также в популяционной выборке (576 лиц, отобранных случайным образом) жителей Западной Сибири. Все обнаруженные варианты вызывали развитие аутосомно-рецессивной формы первичной врожденной глаукомы. При этом наиболее тяжелая клиника наблюдалась у носителей мутаций в 444 кодоне гена. Следовательно, у детей с признаками повышения внутриглазного давления оправдано проведение молекулярно-генетического анализа гена CYP1B1 для ранней диагностики и своевременного начала терапии первичной врожденной глаукомы

## Introduction

Primary congenital glaucoma (PCG, OMIM 231300) is a visual organ pathology that leads to irreversible blindness and poor vision in children. The main cause of PCG is a malformation of the aqueous outflow system and disruption of its filtering ability followed by an increase in intraocular pressure, death of retinal ganglion cells, and as a consequence, blindness or a reduction in visual function (Thau et al., 2018; Badawi et al., 2019). PCG is a rare disease; its prevalence is within the range 1 per 10 000–20 000 live births in the USA, UK, and Ireland and is more frequent (1 per 1250 newborns) in populations where consanguineous marriages are common (Badawi et al., 2019). Most cases of PCG are sporadic, i.e., patients do not have a family history; however, familial cases with autosomal recessive (mainly) and autosomal dominant inheritance have been described as well (Fan, Wiggs, 2010; Souma et al., 2016; Hadrami et al., 2019). Numerous molecular genetic studies have shown genetic heterogeneity of PCG; various genes and combinations of their alleles can be involved in the formation of the pathological phenotype (Liu, Allingham, 2011; de Melo et al., 2015). The predominant cause of the autosomal recessive form of PCG is mutations in the CYP1B1 gene (OMIM 601771): they account for up to 50 % of familial and up to 20 % of sporadic cases (Sarfarazi, Stoilov, 2000). Cytochrome P450 1B1, encoded by the CYP1B1 gene, belongs to the superfamily of enzymes that oxidize steroids, fatty acids, and xenobiotics as well as carry out the biosynthesis of various endogenous compounds (Klingenberg, 1958). The role of this gene in the disease development is still not clear, but it is known that the CYP1B1 monooxygenase participates in the metabolism of retinoic acid, which is essential for normal eye development (Cvekl, Wang, 2009). 

The CYP1B1 gene is located on the short arm of chromosome 2 (2p22.2) and consists of three exons, the first of which is noncoding. A 543-amino-acid protein is encoded by exons 2 and 3 (Vasiliou, Gonzalez, 2008). The polypeptide contains a few functionally significant regions: proline-rich “hinge” and I-helix regions and a cytosolic globular domain, including highly conserved J-helix, K-helix, β-sheets, meander, and heme-binding regions (Stoilov et al., 1998; Zhao et al., 2015).

The enzyme is expressed in many tissues and organs (parenchymal and stromal tissue of the brain, kidneys, prostate, breasts, cervix, uterus, ovaries, and lymph nodes) and in intraocular structures. CYP1B1 mRNA is detectable in the ciliary body, iris, and retina but is absent in trabecular meshwork (Muskhelishvili et al., 2001; Doshi et al., 2006). A study on homozygous mouse knockouts of this gene showed an ocular drainage structure malformation, confirming the involvement of this gene in the development of ocular aberrations (Libby et al., 2003). In humans, CYP1B1 gene expression was revealed throughout all embryonic eye development and during the postnatal period, but its level is higher in fetal eyes than in adult ones. It can be assumed that the product of this gene metabolizes some important substrate that plays a key role in the development and maturation of eye tissues (Doshi et al., 2006).

More than 120 distinct mutations in this gene have been associated with the autosomal recessive form of PCG (http:// www.hgmd.cf.ac.uk/ac/all.php). There are single-nucleotide substitutions in exons and regulatory regions (missense or frameshift mutations or premature stop codons) and larger rearrangements (insertions/deletions) in the CYP1B1 gene, which alter its transcription and translation (http://www.hgmd. cf.ac.uk/ac/all.php). Of note, only one splice site pathogenic variant has been described until now (Afzal et al., 2019). Pathogenic mutations in the CYP1B1 gene associated with PCG are most often localized in the hinge region or the cytosolic globular domain, where they change protein folding, heme binding, and the electron transfer ability (Sarfarazi, Stoilov, 2000).

Thus, the aim of this study was identification of the spectrum of mutations in coding and adjacent noncoding parts of the CYP1B1 gene in patients with PCG in West Siberia by Sanger sequencing.

## Materials and methods

The study protocol was approved by the local Ethics Committee of the Institute of Internal and Preventive Medicine (a branch of the Institute of Cytology and Genetics, the Siberian Branch of the Russian Academy of Sciences, Novosibirsk, Russia). Written informed consent to be examined and to participate in the study was obtained from each patient. For individuals younger than 18 years, the informed consent was signed by a parent or legal guardian

Twenty-eight white patients with PCG were examined. The study population consisted of three families, two patients each (in two families, a pair of monozygous twins and one family with two sisters), and 22 patients who were not related to each other. There were 26 children (10 males and 16 females, from 1 to 12 years old, mean age 8.0±5.5) and two adult sisters aged 45 and 49. All the patients were examined at the Novosibirsk Regional Hospital. Age of onset and a family history were recorded after an interview with the patients or their parents and were based on medical records. All study participants underwent ophthalmic examination: visual-acuity measurement, slit lamp biomicroscopic examination, indirect gonioscopy, tonometry, corneal pachymetry, fundoscopy, and optical coherence tomography of the optic nerve head. Other ocular aberrations and systemic disease were exclusion criteria.

Two control groups were used: healthy and population cohorts. One hundred healthy people (26–83 years old, mean age 67.6±6.9, males 36 %, whites 100 %), who did not have a family history of episodes of glaucoma and other systemic diseases, were enrolled during routine examination at FSBI IRTC Eye Microsurgery (Novosibirsk, Russia). The generalpopulation group (576 subjects total) was randomly selected from two surveys: the population interviewed within the framework of the HAPIEE project (Pajak et al., 2013), Novosibirsk, Russia (376 people, mean age 53.96±6.4 years) and adolescents (188 subjects, mean age 14.83±0.88) from the same region (Zavyalova et al., 2011).

Genomic DNA for Sanger sequencing was isolated from leukocytes of venous blood by phenol-chloroform extraction (Sambrook, Russell, 2006). The primers were reported previously (Gong et al., 2015). PCRs were carried out using BioMaster LR HS-PCR (2x) (BiolabMix, Russia). The cycling program consisted of denaturing at 94 °C for 3 minutes and then 35 cycles of 94 °C for 30 seconds, 68 °C for 30 seconds, and 72 °C for 50 seconds. The PCR products were evaluated by electrophoresis in a 5 % polyacrylamide gel after visualization with ethidium bromide. A 100 bp DNA ladder (SibEnzyme, Russia) was added into each gel as a control. The amplicons were purified on Agencourt AMPure Xp beads (Beckman Coulter, USA), and the sequencing reactions were carried out on an automated ABI 3500 DNA sequencer (Thermo Fisher Scientific, USA) with the BigDye Terminator v3.1 Cycle Sequencing Kit (Thermo Fisher Scientific, USA). The sequences were analyzed in the Vector NTI® Advance software (Thermo Fisher Scientific). We chose a wild-type sequence of the human CYP1B1 gene from the Ensembl Genome Browser (https://www.ensembl.org/index.html) as a reference for alignment.

Rs72549376 and rs377049098 were genotyped by the restriction fragment length polymorphism analysis. Forward and reverse primers were designed by means of the Primer-Blast software (https://www.ncbi.nlm.nih.gov/tools/primer-blast/). The following primers were selected for both single-nucleotide variants (SNVs): 5′-CCTTTATGAAGCCATGCGC-3′ and 5′-TGGTCAGGTCCTTGTTGATGAG-3′. PCRs were set up using BioMaster HS-Taq PCR (2×) (BiolabMix, Russia), 1 μl of each primer, and 1 μl of DNA with a total final volume of 25 μl. The PCR program consisted of initial denaturation at 94 °C for 3 minutes and then 30 cycles of 94 °C for 20 seconds, 59 °C for 20 seconds, and 72 °C for 30 seconds. For genotyping of rs377049098 and rs72549376, 5 U of each restriction enzyme, Tag I and HinfI (SibEnzyme, Russia), were added to the PCR product and incubated for 12–16 h at 37 and 65 °C, respectively.

To detect the C/C genotype of rs377049098, 178 and 43 bp fragments were examined; to detect the T/T genotype, a 221 bp fragment was expected. All the fragments had to be present to detect the C/T genotype. If both gene copies are mutated, then the restriction site is disrupted, and product length should look like that for the T/T genotype: 221 bp. To detect the A/A genotype of rs72549376, 130, 50, and 41 bp fragments were examined; to detect the G/G genotype, 130 and 91 bp fragments were expected. The A/G genotype necessitated the presence of all the fragments. The PCR products were evaluated by electrophoresis in a 5% polyacrylamide gel after visualization with an ethidium bromide solution. A 100-bp DNA Ladder (SibEnzyme, Russia) served as molecular size markers on each gel.

## Results

We analyzed exons and adjacent splice sites of the CYP1B1 gene in 28 patients with PCG. In all the patients examined, the diagnosis of PCG had been made before 2 years of age (in 27 patients before age 1 year); in one male and one female, the damage was unilateral (Table 1). After the diagnosis had been made, 25 patients underwent surgical treatment, and three had a nonsurgical intervention: eye drops. Conservative treatment (selective β-blockers and/or carbonic anhydrase inhibitors) was administered either only as a preoperative preparatory procedure or to prolong the hypotensive effect of antiglaucoma surgery in the late postoperative period (observation period from 1 year to 12 years). In probands P4 and P5, enucleation had been performed due to total retinal detachment, loss of vision, and severe pain syndrome (see Table 1). An analysis of the family history of the patients indicated autosomal recessive PCG inheritance. Genetic analysis of the patients revealed four previously described missense variants R444Q (rs72549376), E387K (rs55989760), R444* (rs377049098), and P437L (rs56175199) and a novel singlenucleotide deletion of cytosine: p.F114Lfs*38 (see Table 1). We did not identify homozygous carriers of a mutation in the studied gene. Compound heterozygous pathogenic variants of CYP1B1 were identified in three PCG cases. In one proband (P1) without a family history of PCG, we identified a single CYP1B1 mutation, R444Q, without any additional genetic variants in this gene. In three families, one patient with PCG was registered (Р1, Р2, and Р3), and in one family, two sisters (Р4 and Р5) turned out to be compound heterozygous carriers of the CYP1B1 substitutions.

**Table 1.**
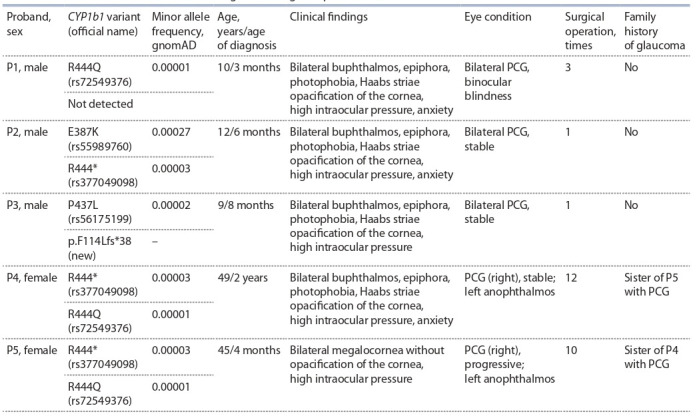
Mutations identified in the CYP1B1 gene among PCG probands No – absent of glaucoma family history

We found no carriers of R444*, R444Q, p.F114Lfs*38, P437L, or E387K among 100 healthy controls. The first two were not found in any of the 576 members of the generalpopulation group. In addition to rare pathogenic variants, we found 6 previously described common SNVs, 5 of which are located in the coding part of CYP1B1 and one in the gene promoter (Table 2).

**Table 2.**
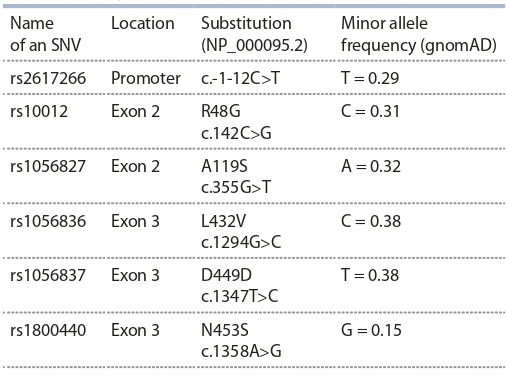
Common variants of the СYP1B1 gene found in West Siberian patients with PCG, and the SNVs’ minor allele frequency according to the gnomAD database

It was previously shown that the detected substitutions (rs10012, rs1056827, rs1056836, rs1056837, and rs1800440) are in linkage disequilibrium in different populations (Chavarria-Soley et al., 2006). For some pathogenic mutations (including E387K, P437L, and R444Q), linkage to certain intragenic haplotypes of the CYP1B1 gene has been shown (Plásilová et al., 1999; Sena et al., 2004; Chavarria-Soley et al., 2006). Accordingly, we analyzed the haplotypes of the CYP1B1 gene in West Siberian patients with PCG that carried rare mutations (Table 3)

**Table 3.**
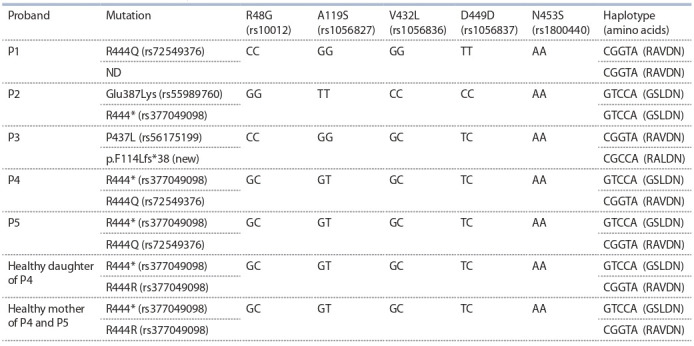
Probable intragenic haplotypes for CYP1B1 in Russian patients with PCG

## Discussion

As a rule, the indications for tonometric examination of children with suspected PCG are symptoms associated with or caused by increased intraocular pressure: pronounced hydrophthalmos, photophobia, tearing eyes, corneal whitening, and anxiety. Because the symptoms may be more or less pronounced, a genetic analysis can confirm the diagnosis, especially in families with previously detected PCG cases. Genetic heterogeneity of PCG makes it difficult to identify causative variants, thereby complicating assessments of disease risk and severity in probands and their relatives. 

Among the analyzed West Siberian patients with PCG, 16 % (four out of 25 unrelated people) were carriers of CYP1B1 mutation(s). Three cases were found to be compound heterozygotes in terms of previously described variants [E387K (rs55989760), R444* (rs377049098), and R444Q (rs72549376)]. In one case, the mutation combination consisted of P437L (rs56175199) and novel frameshift truncating mutation p.F114Lfs*38 (see Figure). 

**Fig. 1.**
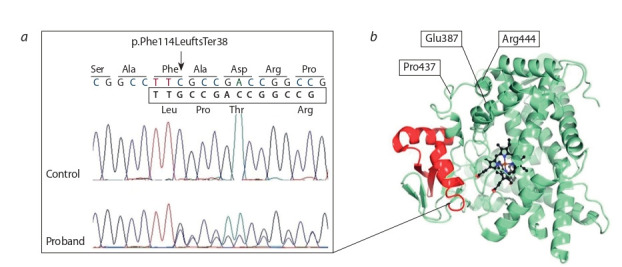
Novel mutation in CYP1B1 gene (a) Аn electropherogram of DNA sequence with the new single-nucleotide deletion, p.F114Lfs*38, in the CYP1B1 gene. (b) 3D-structure of cytochrome P450 1B1 [Protein Data Bank ID 3PM0 (Wang et al., 2011)]. Red means the protein portion remaining unchanged in the case of deletion p.F114Lfs*38; localization of identified amino acid substitutions is indicated.

Amino acid residue Arg444 located in the “meander” region was predicted to be important for structural stabilization of the protein. Replacing Arg with Gln disrupts the contact of the amino acid residue at this position with the oxygen atoms of Trp434 and Asn439 inside the molecule. It can destabilize this structural component and affects the heme-binding and redox functions of CYP1B1 (Mashima et al., 2001). In the case of termination/stop codon Arg444* formation, a truncated protein is synthesized that does not contain the conserved L-helix and heme-binding regions.

In patient P1’s genome, we found only one Arg444Gln substitution in the CYP1B1 gene. Because the proband’s parents did not have glaucoma, his pathological phenotype is most likely due to an additional genetic factor or factors. The same variant in compound heterozygotes was detected in two sisters, P4 and P5. Clinical presentation of congenital glaucoma associated with Arg444 substitution carriage was characterized by faster progression of the pathological process with subsequent loss of vision and an eye as a whole, despite active treatment (12 and 10 operations with subsequent unilateral enucleation in P4 and P5, respectively, and poor vision in the remaining eye; three operations in P1 with binocular glaucoma blindness as a result). Detailed examination of available relatives uncovered no signs of PСG, either in the mother of patients P4 and P5 or in the daughter of P4 (data not shown). Analysis of the general-population and healthy control groups revealed low prevalence of R444Q and R444* among Russians (less than 0.001). 

It is likely that the mutations affecting the “meander” region can aggravate the course of the disease, as compared with other mutations. Mutations in this region have been described previously in Korean, Japanese, Lebanese, and Pakistani patients (Mashima et al., 2001; Chouiter, Nadifi, 2017; Micheal et al., 2017). 

Missense mutation P437L of CYP1B1 has been described previously too. Proline at position 437 is located on the protein surface and determines conformational rigidity of the structure by strongly bending the polypeptide chain. The P437L substitution may alter this special conformation and disrupt interactions of CYP1B1 with other molecules (Rashid et al., 2019). This mutation is found in populations of India, Pakistan, Brazil, Saudi Arabia, and Turkey (Kaur et al., 2011; Chouiter, Nadifi, 2017; Rashid et al., 2019).

The novel single-nucleotide deletion of cytosine in codon 114 of the CYP1B1 gene changes the amino acid sequence and creates a premature stop codon at amino acid position 152. The resulting truncated protein with altered amino acid composition does not contain highly conserved regions, such as the cytosolic globular domain. This variant was not found in gnomAD, HGMD, or ClinVar. Various deletions and duplications in the CYP1B1 gene are described in different populations but are more common in white patients with PCG (Sarfarazi, 2018).

The E387K substitution is most frequent in Europe (Chouiter, Nadifi, 2017). E387 is an invariant amino acid residue for all CYP450 family members (Stoilov et al., 1998; Sorenson et al., 2015). Lysine in this codon disrupts K-helix orientation and prevents formation of a stable hemoprotein complex (Stoilov et al., 1998; Sorenson et al., 2015). Among gypsies in Slovakia, this substitution was found in 100% of PCG cases; this result was explained by the founder effect (Plásilová et al., 1999). Glu387Lys has been identified in French, Brazilian, Canadian, Hungarian, US, and Spanish patients (Melki et al., 2004; Sena et al., 2004).

It was found that among whites, 4 haplotypes are most common for 5 CYP1B1 variants R48G, A119S, V432L, D449D, and N453S. They determine the formation of proteins with amino acids RALDN, RAVDN, RALDS, and GSLDN at the respective positions. Different enzymatic activities were experimentally established for these protein variants (ChavarriaSoley et al., 2008). The enzyme with RAVDN amino acids is 4-fold more active than the variant containing GSLDN. In combination with pathogenic variants, the enzymatic activity is even lower: because of a decrease in the enzymatic activity in the case of G61E and N203S, due to a decrease in the amount of protein in the case of Y81N and E229K, or both in the case of L343del (Chavarria-Soley et al., 2008). Combinations of the polymorphisms and rare mutations can cause additional differences in the phenotypic manifestation of PCG or the severity of the disease. Detection of substitutions R444Q, P437L, and E387K among Russians in the same intragenic haplotypes as in populations from Brazil, USA, Japan, and Romania, and Roma from Slovakia (Chavarria-Soley et al., 2006) allowed us to assume the monophyletic origin of these mutations in Asian, European, Roma, and Brazilian ethnic groups

In Russia, genetic screening of PCG patients has been performed in the Republic of Bashkortostan and in St. Petersburg (14 and 45 patients, respectively) (Motushchuk et al., 2009; Lobov, 2017). No pathogenic substitutions in the CYP1B1 gene were found in the Bashkortostan PCG patients. In one of the St. Petersburg patients, a heterozygous insertion of the CTC trinucleotide in codon 369 (c.1508insCTC, p.P369ins) was detected (Motushchuk et al., 2009); because the family history of the patient was not described in that study, it is impossible to determine the type of PCG inheritance.

Patients with PСG carrying pathogenic variants in the CYP1B1 gene require more surgical operations to correct intraocular pressure and more thorough postoperative maintenance (as compared with patients without mutations in this gene) (Abu-Amero et al., 2011). Therefore, screening for mutations in CYP1B1 gene of children with early-onset glaucoma is advisable for early detection of PCG; such patients subsequently require special attention.

## Conclusion

In white West Siberian patients with PCG, previously described variants E387K (rs55989760), R444* (rs377049098), R444Q (rs72549376), and P437L (rs56175199) and novel frameshift mutation p.F114Lfs*38 were identified. In our study, the most serious clinical phenotype was noted in carriers of mutations R444Q and R444*. Identification of pathogenic variants in patients will contribute to their vision loss minimization owing to early disease detection and regular medical examinations of the substitution carriers. For the patients’ family members, this analysis is also recommended because for individuals who are not carriers of pathogenic variants, the risk of PCG is comparable to that in the general population, and they do not require thorough ophthalmic monitoring.

For verification of PCG diagnoses in West Siberian patients, sequencing of exons and adjacent splice sites of the CYP1B1 gene (rather than searching for point mutations) is recommended due to the absence of major causative mutations.

## Conflict of interest

The authors declare no conflict of interest.
